# Effects of COVID-19 on Adolescent Mental Health and Internet Use by Ethnicity and Gender: A Mixed-Method Study

**DOI:** 10.3390/ijerph19158927

**Published:** 2022-07-22

**Authors:** M. Siyabend Kaya, Ciara McCabe

**Affiliations:** 1 School of Psychology and Clinical Language Sciences, University of Reading, Reading RG6 6AL, UK; m.s.kaya@pgr.reading.ac.uk; 2 Department of Psychology, Abdullah Gül University, Kayseri 38080, Turkey

**Keywords:** COVID-19, adolescent mental health, internet use, ethnicity

## Abstract

Evidence suggests that mental health problems in young people have been exacerbated by COVID-19, possibly related to a lack of social connection. Young people report using the internet for connecting with their peers and mental health support. However, how they may have used the internet for support during COVID-19 is not clear. We wanted to know how mood and internet use may have changed in young people during COVID-19 and if this was different for those with and without depression symptoms. 108 adolescents were recruited. Participants with high and low levels of depressive symptomatology answered questions about their mood, internet use, loneliness and life satisfaction during July and August 2020. We found that the high depression group reported significantly more loneliness and less life satisfaction than the low depression group. We found that most young people used the internet for mental health information during COVID-19 but that the high depression group used the internet more for mental health information than the low depression group. The high depression group also had a worsening of mood compared to the low depression group during COVID-19. We found that Black, Asian and Minority Ethnic participants reported increased use of the internet compared to White participants during COVID-19 and that the role of the family facilitated coping during COVID-19 for some adolescents, but for others, it made the lockdown more difficult. Finally, we found that adolescents perceived school anxiety as stressful as COVID-19. To conclude this study supports the use of the internet as a way to help young people with mental health challenges. It also suggests that the internet is a way to help young people from ethnic minorities, who otherwise might be hard to reach, during challenging times. This study also shows that supportive family units can be important during times of stress for young people and that school anxiety is a major issue for young people in today’s society even outside of the pandemic.

## 1. Introduction

In response to COVID-19, the UK government enforced “lockdowns” to reduce the spread of the virus. The reduction in freedoms changed our way of life [1]. Lockdowns meant the ceasing of all unnecessary travel and social contact outside of one’s own household [2]. These measures combined with the fear of contracting the virus or spreading it to loved ones understandably led to increased mental health difficulties in the population [3]. This increase was also at a time when there was already a large burden on the NHS for mental health services and treatments [4,5]. It was estimated that during COVID-19 the global prevalence of depression was seven times higher than that of pre-COVID [6]. Further, a recent global cross-sectional study with data from 63 countries [7] found that younger vs. older people are more vulnerable to stress, anxiety and depression during the COVID-19 pandemic. Supported by individual studies finding worsening of mood, especially in those 25 years and under during COVID-19 [8,9,10,11]. As adolescence is a critical developmental period for social connections in young people [12] and a period when various psychiatric conditions emerge [13] understandably, the restrictions imposed by COVID-19 may have affected young people more than older people.

It is suggested that up to 75% of young people do not access treatment for their mental health issues [14]. Yet intervening early in a young person’s life could help prevent long-term debilitating mental health issues [15]. As adolescents have widespread internet availability via smartphones, digital interventions could be the way forward to treat otherwise hard-to-reach young people [16,17].

Therefore, in this study, we aimed to examine how young people’s mood and internet use may have been affected by COVID-19. We were also interested to know if young people use the internet more *during* COVID-19 for mental health information. Black, Asian and Minority Ethnic (BAME) groups have faced both physical and mental health inequalities pre-COVID [18] and are disproportionately negatively affected by COVID-19 [19,20]. As calls have been made for specific research into the effects of COVID-19 on ethnic minorities [21] we also wanted to examine ethnicity in our study.

Additionally, we were interested in examining whether mood and internet use during COVID-19 might differ by gender. When compared with boys, girls suffer more mental health problems, especially anxiety and depression [22]. Campbell and colleagues [23] found, in their study of approximately 570,000 adolescents from 73 countries, that girls in every country experience more mental health problems than boys. Contrary to expectations, they stated that this inequality is greater in wealthier and gender-equal countries. Similarly, Fink and colleagues reported that girls’ mental health problems increased while boys’ mental health improved in England between 2009 and 2014 [24]. As studies find that girls are more negatively affected by COVID-19 than boys and their depression and anxiety levels are higher [25,26,27,28] calls have been made for protective measures for the mental health of female adolescents [25,29]. One way to do this might be to harness the avid use of the internet by girls to deliver preventative tools via mobile phone apps [30].

Social isolation caused by COVID-19 has led to a decrease in social relations, individuals feeling more alone [31], and therefore a decrease in life satisfaction [32]. Staying connected is important, especially given that loneliness is thought a possible predictor of adolescent depression [33]. Therefore, in this study, we also examined loneliness and life satisfaction in adolescents [34,35] and hypothesised that young people may have used the internet more during COVID-19 to counteract social isolation and loneliness [36].

During COVID-19, internet and social media sites were used to inform the public and experts about the ongoing situation [37,38]. Studies found that social media could be used as a constructive coping strategy for adolescents to deal with anxious feelings during COVID-19 [39]. Yet there are mixed findings on the impact of social media on mental health in general [40]. Some studies suggest social media can induce stress, depression, loneliness, anxiety, poor sleep, poor well-being, and low self-esteem [41,42]. Whilst a recent longitudinal study found no association between social media use and adolescent depression [43]. Others report positive aspects of social media, especially for maintaining social relations and increasing self-confidence [44]. Therefore, it is plausible that during the lockdown social media activity may have been beneficial for adolescents to keep connected to each other, especially as their mental health was being challenged [8,28,45,46,47,48]. As 95% of adolescents have a smartphone [49] and spend a lot of time on social media, we were interested to know if adolescents used the internet differently during COVID-19 and if this might differ by ethnicity and gender.

Many studies have examined the effects of COVID-19 [26,27,28,50,51,52,53] on adolescent mental health but few have used a mixed-methods approach or examined mood and internet use change [51,52,53]. As we were keen to examine how mood affected internet use and individual experiences of COVID-19, we employed both a quantitative and qualitative approach in line with the recommendations of previous studies [54]. We hypothesised that adolescents with high depression symptoms vs. low depression symptoms might have lower life satisfaction, higher loneliness and increased social media use. We hypothesised that adolescents with high depression symptoms would report a worsening of mood during COVID-19 compared to those with low depression symptoms, use the internet more for mental health information and to keep connected to others. We also hypothesised that ethnic minorities and females would feel their mood had worsened more during COVID-19 than their White and Male counterparts.

## 2. Materials and Methods

### 2.1. Participants

One hundred and eight participants between the ages 16 and 21 years (mean age of 17 years) were recruited via high schools and through poster advertisements. Ethical approval for the study was obtained from the School of Psychology Ethics Committee at the University of Reading. Before testing all participants were sent a link to a webpage with the study information and a consent form to sign. All participants received £10 reimbursement for their time. We compared responses in participants (*N* = 65) with high levels of depressive symptomatology (HD; Mood and Feelings Questionnaire, MFQ ≥ 29; [55,56] to participants (*N* = 43) with low levels of depressive symptomatology (LD; MFQ scores < 29). The MFQ has been reported as having Cronbach’s alphas of 0.91 and above in a New Zealand sample, indicating excellent internal consistency [57]. The MFQ Cronbach’s alpha for the data in this study was 0.93. For more on MFQ psychometrics see https://www.corc.uk.net/outcome-experience-measures/mood-and-feelings-questionnaire-mfq/ (accessed on 10 July 2022).

### 2.2. Questionnaires

Participants completed demographic questions about age, gender and ethnicity. They also completed the Mood and Feelings Questionnaire (MFQ) [55] (high scores indicate greater depression symptomatology), the University of California Los Angeles (UCLA) Loneliness Scale (a high score indicate greater feelings of loneliness) [58], the Satisfaction with Life Scale (SWL, high scores indicate a higher life satisfaction) [59] and the Social Media Use Integration Scale (SMUIS, a high score reflect more engaged use and integration of social media) [60]. Participants also answered the following four questions about mood and internet use (Table 1). For the qualitative section of the survey, we devised open-ended questions about how lockdown affected mood and internet use (Table 2).

Researchers [61] state that the number of people in such studies can vary between five and twenty-five. Thirty-two participants that reflected the typical characteristics of the group [62] were selected for further qualitative interviews about their moods and internet use [63]. Using criterion-based sampling [63], we took the MFQ mean of the LD group and the HD group and included individuals closest to this average score and found an equal representation of gender and ethnicity across the depression groups. This resulted in 16 BAME and 16 White participants, of which there were 4 males and 4 females with HD and 4 males and 4 females with LD. The BAME group consisted of equal numbers of Black and Asian participants.

### 2.3. Quantitative Analysis

Using SPSS (IBM SPSS Statistics for Windows, Version 25.0. Armonk, NY, USA), we used a mixture of parametric and non-parametric statistics to assess the differences between the HD and LD groups. When assessing the scores for mood change, we re-coded the scores from −5 to +5 to account for both positive and negative changes.

### 2.4. Qualitative Analysis

Using NVivo 12.0 (QSR International Pty Ltd., Doncaster, Australia) we performed content analysis to code and categorize the patterns, frequencies and relationships in the answers to the open-ended questions [64]. We report the codes and categories in the text and the quantified qualitative data in the appendices. The comparisons we made between groups in the text are based on the content analysis of the data given in the appendices. Reading all participants’ interview transcripts (tree-coding process) we transferred the codes into more comprehensive codes and then examined the relationship between the codes to create final themes [65]. Thus, we followed a logical inductive approach to form meaningful and holistic themes. We frequently use direct quotations in the text to reflect the expressions of the participants more clearly and abbreviations to indicate the ethnicity (BAME or White), gender (Male or Female) and depression grouping (HD for High depression symptoms or LD for Low symptoms).

### 2.5. Trustworthiness

**Figure 1:**
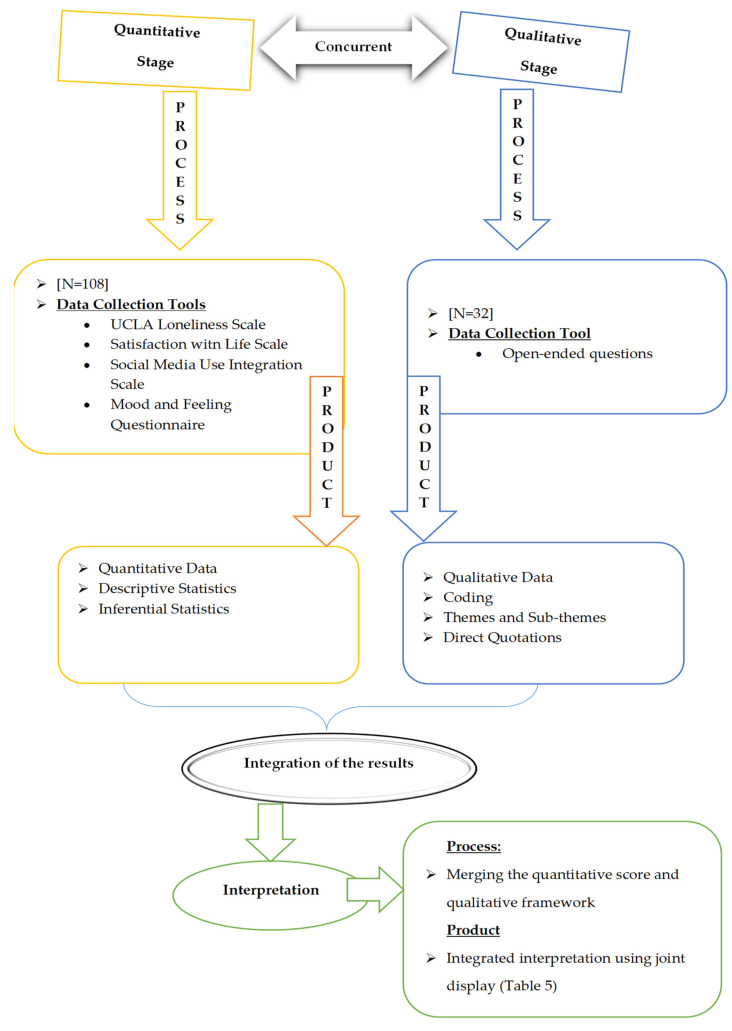
The process diagram of the research.

We used several strategies to build up trustworthiness in the qualitative part of the study [66]. For example, we received support from another academic to evaluate the study from a different and critical perspective as proposed by Lincoln and Guba [67]. We also had two coders and checked the intercoder reliability (ICR) and report a Cohens kappa coefficient [68,69] of 72 and ICR to be 86% using Miles and Huberman’s [65] formula. We used a convergent parallel design (mixed-methods) [63,70]. After independently analysing the quantitative and qualitative data we merged the data using a “joint display” comparison method [71] to show convergence and divergence. The process diagram of the research is given in Figure 1.

## 3. Results

### 3.1. Quantitative Results

This We found no differences in gender (χ^2^(1 108) = 2.92, *p* = 0.08) ethnicity (χ^2^(1 108) = 0.64, *p* = 0.42) or education (χ^2^(2 108) = 2.6, *p* = 0.26) between the HD and LD groups. We did find a difference in HD and LD use of the internet for mental health information (χ^2^(1 108) = 21, *p* < 0.001) with 92% of HD vs. 53% of LD answering yes to using the internet for mental health information in line with our hypothesis. We also found significantly more females answered yes to using the internet for mental health information than males, 82% vs. 56% (χ^2^(1 108) = 7.446, *p* = 0.006). Also, we found no statistical difference in the effect of ethnicity on using the internet for mental health information with 81% White and 69% BAME responding yes. Using an independent samples t-test we found significantly higher Life Satisfaction scores in the LD vs. the HD group as might be expected but no difference on the Social Media Use questionnaire (Table 3). There were no gender or ethnicity differences on the questionnaires for Social Media Use and Life Satisfaction scores.

For the data that was not normally distributed, we used the Mann-Whitney *U* test and found increased loneliness (UCLA) in the HD group compared to the LD group and a worsening of mood during COVID-19 in the HD group vs. the LD group as hypothesised. We also found increased use of the internet during COVID-19 and increased internet use for mental health information in the HD vs. the LD group (Table 4) as hypothesised. When examining gender and ethnicity we found increased use of the internet during COVID-19 in BAME compared to White participants (*U* = 4.45, *p* = 0.03).

**Figure 2:**
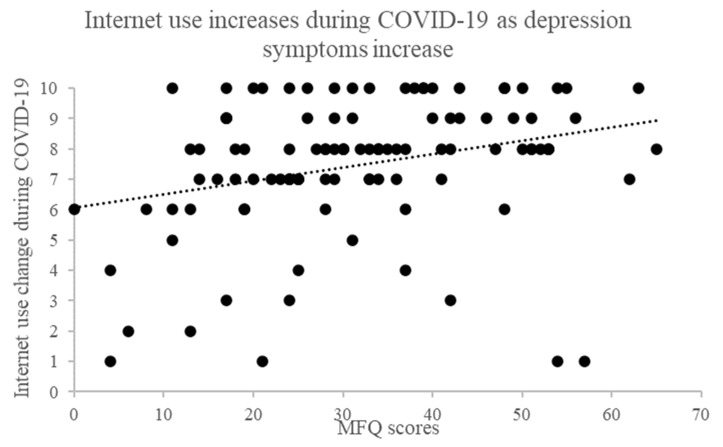
Correlation between MFQ and internet use during COVID-19.

**Figure 3:**
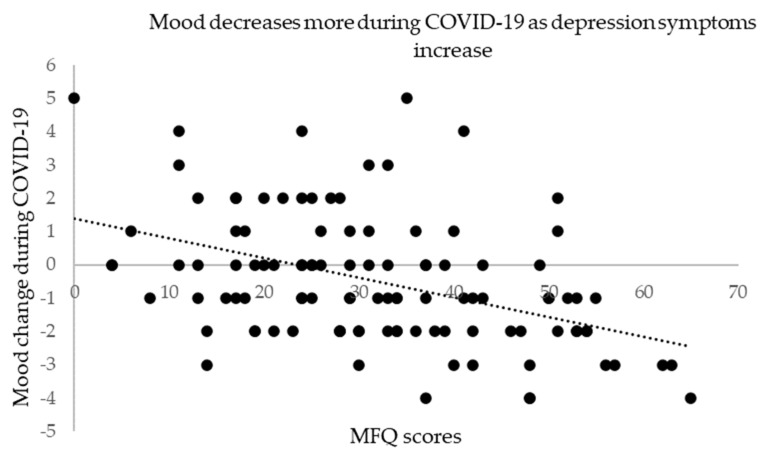
Correlation between MFQ and mood change during COVID-19.

We performed Spearman’s correlation test to assess the relationship between depression symptoms (MFQ) and the 4 quantitative survey responses. Results of *p* ≤ 0.01 were considered significant to control for multiple comparisons. Consistent with our between-group analysis we found a statistically significant positive correlation between internet use change during COVID-19 and MFQ scores, (r_s_ = 0.34, *N* = 108, *p <* 0.001, two-tailed, see Figure 2) that is those with higher depression symptoms reported using the internet more during COVID-19. We also found using a Pearson’s correlation test that those with higher depression symptoms had a greater decrease in mood during COVID-19 (r = −0.44, *N* = 108, *p <* 0.001, two-tailed, see Figure 3).

### 3.2. Qualitative Results

Content analysis of the experiences expressed by the adolescents to the qualitative questions revealed 3 themes:

(1) The effect of internet use on mental health during COVID-19;
(2) Mood and life satisfaction before COVID-19;
(3) Mood and life satisfaction during COVID-19.

The results are summarised below with some verbatim quotes taken from participants.

As highlighted earlier, quantifying the qualitative data is a method used in content analyses that facilitates comparisons between themes/groups by calculating the percentage or frequency of participants’ statements about the related theme(s). See the appendices for quantified qualitative data.

(1) The effect of internet use on mental health during COVID-19

**Figure 4:**
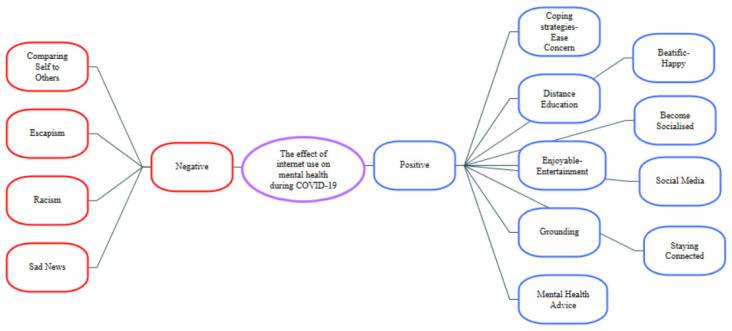
Themes/Sub-themes regarding the effect of internet use on mental health during COVID-19.

Two main themes emerged: “positive” and “negative” aspects. Themes were created in line with the statements of the participants (see Figure 4).

Results show that across all participants positive aspects of internet use on mental health during COVID-19 were reported more than negative aspects. One of the participants summarises this as follows:

*“I actually think the internet has improved my mood. My friend created a new group chat on Instagram, I have enjoyed talking with everyone. I believe that without this chat I would have found it difficult to reach out to people and would’ve begun to isolate myself. It has stopped me from feeling lonely. Furthermore, the internet has provided me with good entertainment to make my lockdown better. The only negative that the internet has brought is the constant stream of upsetting news stories, I found myself having to ignore information such as COVID-19 statistics to prevent my anxiety.”*

(13WHITE-LD-F)

Another participant summarised almost all positive aspects of internet use as follows:

*“I have used the internet more than usual and it has in some aspects positively affected my mood. I have made more online friends with whom I communicate with regularly and share my schoolwork and social life with. For leisure, I have also, used my play-station to play online games with other friends. I have used the internet significantly more for both educational and social purposes. Educationally, I have used the internet to research topics relating to COVID-19 as well as mental health issues related to the elderly during the pandemic. This has helped me to better understand and take care of my grandmother. Socially, I have interacted with other people with who I would otherwise have not shared my views.”*

(51BAME-LD-M)

When examining the effects of depression, we found that those in the HD group emphasized the positive side of the internet more than those in the LD group (see Appendix A):

*“I’ve used the internet a lot more, for communication and entertainment purposes. I also seek mental health advice online when I can, and regularly check the status of COVID-19 to ease my concerns, such as what the government has said, the rates of it, scientific advice etc.”*

(18BAME-HD-F)

When looking at the effects of internet use on mental health during COVID-19 and ethnicity and gender we found that White participants emphasized the negative aspects of the internet more than the BAME group and females emphasized the negative aspects of the internet more than males.

Here is an excerpt, from a White female participant, stressing the negative impact of the internet on mental health during COVID-19. This was expressed as individuals comparing themselves with others and feeling the need to escape from seeing others do things online e.g., *“Decreased my sense of self-worth, started comparing myself to people who were very active during lockdown where all I wanted to do was sit and watch tv (as an escapism I guess). I compared myself to the people who were doing work, working out and trying out new skills. I also spent a lot of time comparing my body to others on social media.”* (100White-HD-F)

(2) Mood and Life Satisfaction Before COVID-19

**Figure 5:**
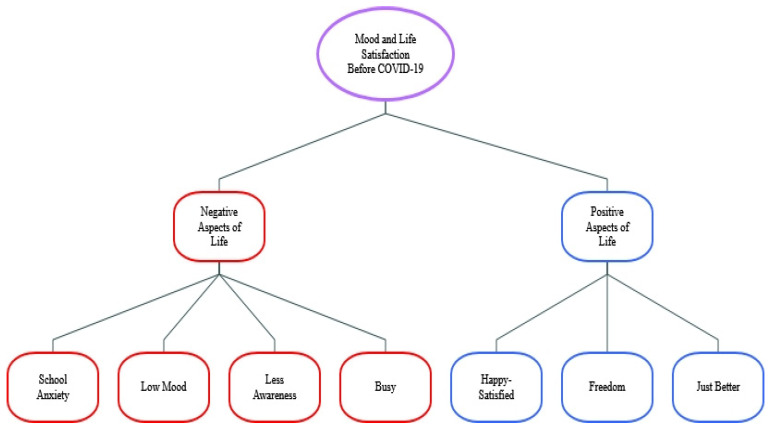
Themes/Sub-themes for mood and life satisfaction before COVID-19.

For the theme, mood and life satisfaction before COVID-19, two sub-themes emerged: positive and negative aspects. Freedom and happiness were further positive sub-themes and school anxiety, low mood and being busy negative sub-themes (see Figure 5).

When asked about mood and life satisfaction before COVID-19, participants’ reports formed two main themes positive and negative aspects. Participants however emphasized more frequently the negativities they experienced. Participants reported for example that school and exams impacted their mental health:

*“My mood before the lockdown was much brighter and happier but I was a bit more stressed with school. Now my mood is less bright more drowsy and fatigued but less stressed. That’s all.”*

(2BAME-LD-F)

*“I felt more peaceful and calm because not going to school decreased my social anxiety since I could stay home.”*

(69White-HD-M)

*“Due to not having to be at school, my fear and stress have decreased a lot.”*

(72White-HD-F)

When asked about mood and life satisfaction before COVID-19 and comparing those with high and low depression symptoms we found that the HD participants focused on negative aspects more, for example:

*“Before lockdown, my mood was low often and my life satisfaction was low also. Due to school and exams, I had no free time nor did I have time to think about my mental health as all my focus was on achieving good grades and getting homework done.”*

(18BAME-HD-F)

Compared to White participants, BAME participants reported positive aspects more, for example: *“Before the lockdown, I had more direct face-to-face social contact with many of my friends which I enjoyed very much. However, there were times when I wanted to have some ‘me time’.”* (51BAME-LD-M)

Finally, we found that Female participants focused on positive aspects more than males, for example:

*“My mood before lockdown was much better. I think having a daily routine and a plan helped me a lot but since lockdown, it’s thrown me. Being able to spend time face to face with my friends even at school was something that I really enjoyed. I think my satisfaction has gone down a little but is maintained from me being active and taking part in a variety of activities.”*

(48BAME-LD-F)

(3) Mood and Life Satisfaction During COVID-19

**Figure 6:**
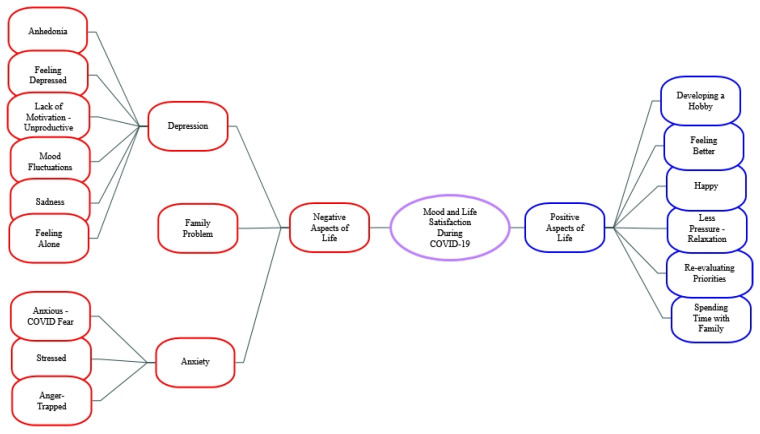
Themes and Sub-themes regarding mood and life satisfaction during COVID-19.

When asked about mood and life satisfaction during COVID-19 again two main themes of positive and negative aspects emerged (see Figure 6).

Across all participants, negative aspects of mood and life satisfaction during COVID-19 fell into 3 sub-themes: Depression, Anxiety and Family problems. Depression was the sub-theme most emphasized by participants. For example: *“Since lockdown, being away from my girlfriend for months left me feeling very trapped and depressed and led to a general feeling of loneliness throughout the lockdown period.”* (80White-HD-M)

Examples of anxiety as a negative aspect:

*“Initially the start of lockdown was not great, I felt rundown by the building pressures at school prior to lockdown and suddenly being isolated from others brought on a lot of stress.”*

(37BAME-LD-F)

Other participants reported a kind of flattening of moods and life satisfaction during COVID-19 with negative aspects of uncertainty:

*“…the monotony of lockdown, it’s such a contrast from my usual daily life that it’s almost confusing to me. It has made my mood weirdly stable, and my range of emotions seems more limited than before. Each day seems to have merged into each other. I can’t pinpoint anything that has given me an overwhelmingly "good" or "bad" mood. There has been a couple of times where I have been upset about the uncertainty of the future and feeling that I’m wasting my time.”*

(13White-LD-F)

*“Although my mood is ‘good’ I have found that my life satisfaction has decreased. I look back on the last few months and I can’t think of any memorable events or times where I had a great day. It almost feels like lost time, like I’ve been stuck in an airport since March.”*

(13White-LD-F)

When asked about mood and life satisfaction during COVID-19, participants also described both positive and negative aspects concerning family relationships. The expression below exemplifies this

*“I think that my family have affected my mood the most in a bad way. I think I am generally grumpier and more fed up and I snap a lot due to being around only them. But on the other hand, I think talking to my friends has affected my mood in the best way, as I don’t often have long conversations with my friends, recently I have had more and have enjoyed them.”*

(32BAME-HD-F)

When asked about mood and life satisfaction during COVID-19 and comparing those with high and low depression symptoms we found that the LD participants had higher life satisfaction and could see life more positively than HD participants:

*“I felt more awake and relaxed, as I didn’t have major exams to stress about anymore. I am getting the right amount of sleep for myself, and I was able to pursue my hobby of making bracelets. It did get a little stressful as I found it harder to do schoolwork at home. Overall, I was happier during lockdown than before lockdown.”*

(76White-LD-F)

When asked about mood and life satisfaction during COVID-19 and comparing ethnicities there was no marked difference between ethnic groups. We did observe, however, female participants emphasized positive aspects more than males (Appendix B). For example:

*“Due to how much school negatively affected me, my mood initially spiked and I have felt happier and more in control since. However, the worry of going out and worrying about vulnerable family members, not being able to see people and the isolation has negatively affected how I feel, particularly about myself. However, overall, the break to my normally busy life has done better than anything.”*

(72White-HD-F)

### 3.3. Merged Analysis Results

We compared qualitative themes with the quantitative findings and merged these two data sets as shown in Table 5. During the data integration phase, we observed that the qualitative data mostly confirmed the quantitative data. For example, qualitative findings confirmed that HDs feel lonelier and have lower life satisfaction than LDs. Also merged data confirmed that females compared to males use the internet more for mental health information.

Also, it is important to report where the qualitative data expanded the quantitative. For example, during the quantitative analysis, we found that the HD had overall increased internet use and specifically increased internet use for mental health information, whilst the qualitative analysis revealed that across all participants the advantages of using the internet during COVID-19 were frequently reported. Further, the qualitative data captured information about mood and internet use that was not otherwise apparent such as the effects of school/exam anxiety being very stressful for adolescents. Furthermore, the qualitative data revealed the effects of family relationships during COVID-19 and their effects on adolescent mental health.

## 4. Discussion

This study set out to explore internet use and mood changes during COVID-19 in adolescents with high and low depression symptoms. We found higher levels of loneliness and lower levels of life satisfaction, in those with high depression symptoms compared to low symptoms, as might be expected. As loneliness can increase the risk for depression and can worsen depression for those already depressed [72], it is important to recognise any additive effects COVID-19 may have had on those with depression symptoms or those at risk of clinical depression.

As hypothesised, we found that mood worsened in those with high depressive symptoms compared to low depressive symptoms during COVID-19. Perhaps, as a way to counteract loneliness and social isolation, we found that during COVID-19 adolescents increased their amount of time on the internet and those with high depression symptoms increased their time the most. Furthermore, 87% of young people in this study and 92% of those with high depression symptoms said that they used the internet for mental health information and that this increased during COVID-19, especially for those with higher depression symptoms. This emboldens the view that digital technologies can be harnessed for youth mental health support [73,74]. Our data thus provides new evidence that during COVID-19 young people were using the internet for support despite any decreases in mood and social connection. This is encouraging as new avenues for mental health treatment are sorely needed that can help penetrate the lives of hard-to-reach young people [16,17]. Moreover, as 53% of those with low depression symptoms also reported using the internet for mental health information this data shows that the internet can also be used as a preventative tool for mental health struggles [75].

Apart from looking for mental health information, the qualitative results help us understand how young people experience the internet with regard to their mental health. For example, we found adolescents used the internet in a positive way such as by surfing social media, keeping up to date with current information, continuing their (distance) education, and having fun. This extends other recent research showing that adolescents stand to benefit from using the internet for connecting with support systems, discovering accurate information, and even taking a break from the stressors of everyday life [76]. However, participants also mentioned how the internet negatively affects them by being exposed to sad news for example. Consistent with other studies, young people also reported the internet as negative because they use it to compare themselves with others [77,78,79]. Similar to previous studies [80] participants also reported the internet as escapism. This could be seen as both positive and negative in that using the internet to escape reality/everyday stressors might help one’s mood in the short term. However, escaping issues, that require dealing with in the short-term, could lead to larger longer-term problems.

In answer to the qualitative questions, adolescents reported that school anxiety before COVID-19 was just as stressful as COVID-19 itself. This is consistent with other reports that adolescents were particularly worried about their schooling during lockdown [81]. However, as this data was recorded during the most intense period of COVID-19, it is worrying that students were as anxious about school as COVID-19. Studies [82,83,84] report the negative effects of school anxiety on adolescents, yet this is the first study, to our knowledge, to show that in their own words some young people found COVID-19 a welcome relief from school and the anxiety it causes.

Young people in this study also reported that good family relationships can help them cope better with situations like COVID-19 whilst bad family relationships can make it worse. For example, families being too strict and arguments about studying are reported as reasons for mood decreases in young people. We should emphasise that the literature on this subject is not very consistent. Some researchers [81] show that time spent with family during COVID-19 has a positive effect on adolescent life satisfaction, while others [85] emphasise the opposite (especially in cases of domestic abuse and violence). Therefore, our study adds some understanding about which family situations contribute positively or negatively to adolescent mental health.

Regards gender, we found that females reported using the internet for mental health information more than males which may reflect the higher prevalence of depression symptoms in females in society and fits with the data that adolescent females use the internet more than males [49]. We also found during the qualitative part of the study that more males than females reported school anxiety as worse than COVID-19 anxiety, but this was only a few participants. Further, more females compared to males reported negative experiences with being online, which could be explained by increased use of the internet for social media in females vs. males [86] rather than gender effects per se as it has been hard to show this in other studies [87].

Regards ethnicity we found that Black and Asian minority ethnic adolescents reported increased use of the internet during COVID-19 compared to White adolescents. We also found during the qualitative part that BAME participants reported less negative and more positive aspects of being on the internet compared to White participants. This is promising new evidence that the internet can be used for interventions for minority groups that may be struggling with mental health issues. Especially as ethnic minorities have been shown to be differentially affected by COVID-19 [19,20,21], are more likely to suffer from depressed moods compared to White participants [88] and are less likely to ask for help [89].

Taken together, our mixed-methods study allowed us to examine adolescents’ experiences of mood and internet use during vs. before COVID-19. Most studies on internet use and mood in adolescents use forced-choice questions thus our use of qualitative questions with open-ended answers increased our understanding of the adolescent experience. The diversity of participants and researchers’ was also a strength of this study and we were able to examine both ethnicity and gender effects in our data.

## 5. Conclusions

In summary, we have shown that those with higher depression symptoms used the internet more for mental health information and had worsening of mood during COVID-19 than those with lower depression symptoms. We have shown that school anxiety is something adolescents report as being as stressful as COVID-19 itself and that adolescents with low depression symptoms also used the internet for mental health information during COVID-19. This supports the view that the internet can enable adolescents, with a range of depression symptoms, to get mental help support. We have shown that ethnic minority adolescents used the internet more during COVID-19 than before COVID-19 and that females emphasized the negative consequences of the internet more frequently than males but also used the internet more often to seek mental health information.

Regards limitations, we had a small sample size for the quantitative analysis and no questions about what participants were doing online, but we did then capture this in the rich qualitative data. Not collecting the nationality of participants could also be considered a limitation as it is possible that there might be differences within White or BAME experiences depending on nationality. Therefore future studies could examine with a combination of face-to-face and online interviews the intersectionality of ethnicity, nationality and gender in relation to depression symptoms and internet use in young people.

## Figures and Tables

**Table 1:** Quantitative questions.

1. Have you used the internet for mental health information? yes or no
2. If yes, how has your internet use for mental health information changed during the lockdown? on a scale of 1 to 10, 1: decreased; 10: increased.
3. How has your internet use changed during COVID-19? on a scale of 1 to 10, 1: no change; 10: much increased.
4. How has your mood changed during COVID-19? on a scale of 1 to 10, 1: much worse; 10: much improved.

**Table 2:** Qualitative questions.

1. How has lockdown affected your mood? Can you give any examples?
2. How has the internet affected your experiences during the lockdown, especially your mood? Can you give an example?
3. Have you used the internet more during lockdown? If so what for?
4. Have you used the internet more during lockdown for mental health information?
5. Have you used the internet more during lockdown for information about COVID-19?
6. When you compare your mood/life satisfaction before and during the lockdown, what would you like to say?
7. Since lockdown what has affected your mood the most? Good or bad? Can you give any examples?

**Table 3:** Descriptive and inferential statistics for participants with low and high depression scores.

	LD (*N* = 47) vs. HD (*N* = 61)		Group Difference	
Nominal Data	Count		χ^2^	*p*-Value
**Gender (F/M)**	30/17	48/13	2.92	0.08
**Ethnicity**				
(BAME/White)	26/21	29/32	0.64	0.42
**Education**			2.6	0.26
Secondary	2	5		
College	39	53		
University	6	3		
**Use of the Internet for Mental Health Information**			21	<0.001
Yes	25	56		
No	22	5		
	**LD (*N* = 47) vs. HD (*N* = 61)**		**Group Difference**	
**Norm Distributed Data**	**Mean (SD)**	**Mean (SD)**	***t*** **Score**	***p-*** **Value**
MFQ	18.68 (7.06)	41.97 (9.74)	−13.8	<0.001
SWL	22.30 (5.84)	17.59 (6.20)	4.0	<0.001
SMUIS	37.17(8.23)	39.18 (11.27)	−1.0	0.306

**Table 4:** Mann Whitney-*U* test results between LDs and HDs.

Non-Norm Data LD vs. HD	Mann-Whitney *U*	*p*-Value
Age	1333.5	0.43
UCLA	2245	<0.001
Mood changes during COVID-19	844	<0.001
Internet change COVID-19	1975	0.001
Internet mental health info change	938.5	0.01

**Table 5:** Merged analysis of quantitative and qualitative data.

Main Theme	Quantitative Results (Statistics)	Findings Based on Qualitative Observation	Meta-Inferences (Interpretation)
**Impact of internet use on mental health**	HDs use internet more for mental health information (χ^2^(1 108) = 21, *p* < 0.001) than LDs. BAME adolescents reported increased use of the internet more than White adolescents (*U* = 4.45, *p* = 0.03). Those with increasing symptoms reported using the internet more during COVID-19 (r_s_ = 0.34, *N* = 108, *p* < 0.001, two-tailed). Furthermore, as depression symptoms increased participants were more likely to use the internet for mental health information (r_s_ = 0.37, *N* = 108, *p* < 0.001, two-tailed)	All participants: *Since lockdown, my mood has been most affected by social media, positively, I’ve made new friends and found new hobbies, such as video editing and knitting, which I never thought I’d enjoy prior to seeing it online! My new online friends, I found I could confide in better than my in-person friends which were also helpful.* (18BAME-HD-F)	**Confirmation** Both groups indicated that they used the internet more for mental health at the quantitative stage and reported positive aspects of the internet for mental health at the qualitative stage.
**Mood and life satisfaction before COVID-19**	No quantitative data before COVID-19	Participants emphasized that school anxiety and an intense pace of life made them unhappy before COVID-19: *My mood has increased massively as I was really tired of waking up early and travelling to school.* (12BAME-HD-M) *Well, the lockdown helped my social anxiety a lot in the sense that I didn’t have to go to school and be in a crowded environment, so I was able to concentrate more on studying and be calmer.* (69White-HD-M)	**Expansion** The statements taken from the participants regarding COVID-19 in the qualitative phase contributed to the creation of a new main theme “pre-COVID-19”. However, in the quantitative stage, no measurement tool can directly measure the pre-pandemic period, so quantitative data on this subject could not be obtained. However, the mixed-method research fulfilled its function and contributed to revealing such an important finding that could not be reached at the quantitative stage.
**Mood and life satisfaction During COVID-19**	HDs have lower life satisfaction *t*(106) = 4.01, *p* < 0.001 and higher loneliness levels (U = 2245, *p* < 0.001). As depression symptoms increase, mood during COVID-19 decreased (r = −0.44, *N* = 108, *p <* 0.001, two-tailed).	As expected, the participants emphasized that COVID-19 negatively affected their life satisfaction: *My mood and life satisfaction have always been high, and I smile to make those around me smile too and be happy. There is no perfect life, so I cannot say that it is or isn’t perfect but due to the changes that have occurred due to COVID-19 there have been changes in my mood, I am still happy, but I worry about those who are close or even those that I do not know.* (87BAME-LD-M)	**Confirmation** During COVID-19, it is observed that adolescents experience deterioration in their mood. Participants expressed the negative effects of COVID on mood more frequently, and this confirms the data obtained from the quantitative stage.
**Mood and life satisfaction During COVID-19**	There was no statistically significant difference between genders regarding life satisfaction (*p* > 0.05).	Male participants pointed out the negatives of COVID-19 more than females did: *My mood definitely took a sharp decline during the lockdown period due to the lack of outside interaction and the feeling of being trapped and isolated from the outside world in comparison to pre lockdown where all was good in the world and my mood has generally quite good.* (80White-HD-M)	**Discordance** Although life satisfaction and loneliness levels were not statistically different between males and females, the qualitative stage of the study revealed that males have a more negative perspective compared to females.
**Mood and life satisfaction During COVID-19**		Negative relationships with the family could have caused negative aspects on adolescents’ mental health: *My energy levels have just decreased significantly. I have more arguments with my family members in the same household. I have felt happy, sad, and I’ve cried a few times. Sometimes, I cry for no reason sometimes I cry because I have a reason. it has also made me think about my body and figure a lot more and my physical appearance.* (46BAME-LD-F)	**Expansion** The effect of family relationships on adolescents’ moods during COVID-19 was revealed in the qualitative part. Families stand out as an important factor that facilitates coping with COVID-19 but for some, families made COVID-19 more difficult.

## Data Availability

Data cannot be shared publicly because participants did not consent to the data being made public. Data are available from the School Ethics Committee, School of Psychology and Clinical Language Sciences, Harry Pitt Building, Whiteknights Campus, The University of Reading, Reading, RG6 6AL, UK, for researchers who meet the criteria for access to de-identified confidential data.

## Appendix A

**Table A1:** The effects of gender, ethnicity and depression level on internet.

Codes/Categories	Gender		Depression Level		Ethnicity	
	Male	Female	HD	LD	White	BAME
Negative	4%	14%	11%	8%	11%	7%
Comparing self to others	1%	4%	3%	3%	4%	1%
Escapism	0%	4%	2%	3%	2%	3%
Racism	2%	1%	3%	1%	1%	2%
Sad news	1%	4%	4%	2%	4%	1%
Positive	41%	41%	46%	36%	36%	46%
Beatific	3%	3%	4%	2%	1%	5%
Become socialised	4%	3%	4%	4%	3%	4%
Coping strategies-Ease Concern	4%	4%	6%	3%	3%	6%
Distance education	3%	4%	2%	6%	4%	4%
Enjoyable-Entertainment	6%	4%	6%	4%	4%	6%
Grounding	7%	10%	10%	7%	8%	8%
Mental health advice	2%	3%	2%	3%	1%	4%
Social Media	4%	4%	6%	3%	4%	4%
Staying connected	7%	4%	6%	5%	7%	4%
**Total**	**45%**	**55%**	**57%**	**54%**	**47%**	**53%**

Since the study group of the study was taken in equal numbers from each demographic variable (16 BAME, 16 White; 16 Female, 16 Male; 16 HD, 16 LD), the percentage calculation was made over the whole number of coded expressions under each demographic variable. For example, since the number of coding received from males and females (under the gender demographic variable) was 157 in total, the percentage of codes that females express (22 expressions) as negative is calculated as 22/157 × 100 = 14%.

## Appendix B

**Table A2:** The effects of COVID (before and after) and gender and ethnicity on mood and life satisfaction.

Codes/Categories	Gender		Depression Level		Ethnicity	
	Male	Female	HD	LD	White	BAME
**A-Before lockdown**						
**A1-Negative Aspects of Life**	38%	31%	41%	28%	38%	31%
Less awareness	5%	0%	5%	0%	5%	0%
Low mood	0%	5%	3%	3%	3%	3%
No free time	3%	3%	3%	3%	0%	5%
School anxiety- School worse than COVID	31%	23%	31%	23%	31%	23%
**A2-Positive Aspects of Life**	15%	15%	18%	13%	13%	18%
Better	5%	5%	3%	8%	3%	8%
Freedom	5%	3%	5%	3%	3%	5%
Happy-satisfied	5%	8%	10%	3%	8%	5%
**Total**	**53%**	**46%**	**59%**	**41%**	**51%**	**49%**
**B-During lockdown**						
**B1-Negative Aspects of Life**	36%	30%	37%	29%	32%	34%
Anger-Trapped	3%	1%	2%	2%	3%	1%
Anhedonia	1%	2%	1%	2%	2%	1%
Anxious-COVID fear	2%	4%	5%	1%	4%	2%
Feeling Depressed	3%	3%	5%	1%	2%	4%
Difficult to cope with	0%	2%	1%	1%	1%	1%
Family problems	3%	3%	2%	4%	2%	4%
Lack of motivation-Bored-Unproductive	5%	3%	4%	4%	4%	4%
Loneliness	3%	2%	3%	2%	2%	4%
Mood fluctuations	3%	2%	2%	3%	3%	2%
Restricted-isolated	7%	5%	7%	5%	5%	7%
Sad	1%	3%	2%	2%	2%	2%
Stressed	2%	2%	2%	2%	2%	2%
Worse	3%	2%	3%	2%	3%	2%
**B2-Positive Aspects of Life**	11%	23%	14%	20%	16%	18%
Developing a hobby	2%	4%	1%	4%	2%	4%
Feeling better	2%	6%	3%	5%	4%	4%
Happy	3%	4%	2%	4%	4%	3%
Increasing awareness	2%	3%	3%	2%	3%	2%
Less pressure-Relaxation	1%	3%	2%	3%	2%	2%
New friends	1%	1%	1%	0%	0%	1%
Re-evaluating priorities	1%	3%	2%	2%	1%	3%
Spending time with family	1%	1%	0%	2%	2%	0%
**Total**	**47%**	**53%**	**51%**	**49%**	**48%**	**52%**
